# A CROSS-NATIONAL COMPARISON OF THE EFFECT OF SMOKING AND DRINKING BEHAVIOR ON COGNITIVE HEALTH IN TAIWAN AND THE US

**DOI:** 10.1093/geroni/igae098.2477

**Published:** 2024-12-31

**Authors:** Yan-Jhu Su, Yao-Chi Shih

**Affiliations:** University of Massachusetts Boston, Boston, Massachusetts, United States; National Taipei University of Nusring and Health Sciences, Taipei, Taipei, Taiwan (Republic of China)

## Abstract

Previous studies have shown that smoking tobacco and alcohol consumption impact individuals’ cognitive health. However, less studies have focused on cross-national comparisons of the effects of smoking and alcohol consumption on cognitive health and their differential effects across gender. This cross-sectional study used the Health and Retirement Study (HRS) 2018 (N= 7,467) and the Taiwan Longitudinal Study in Aging (TLSA) 2019 (N=2,776) for comparison. The cognitive function scores of the HRS are from the Telephone Interview of Cognitive Status-Modified (TICS-M; range: 0-35). For the TLSA, the Montreal Cognitive Assessment (MoCA; range: 0-30) is used. Our results show that both smoking and drinking negatively affect one’s cognitive health among the US and Taiwanese populations, yet important gender differences were found. Taiwanese women aged 60 and older were found to have lower cognitive scores relative to otherwise similar men; however, in the United States, middle-aged and older women were found to perform better in their cognitive tests than their male counterparts. No moderating effects of gender on smoking were found among older persons from both countries. Some drinking was found to be protective for Taiwanese women, while binge drinking among US women further negatively impacted their cognitive health. Findings suggest that both smoking and drinking may impose negative influence on one’s cognitive health and these impacts differ by gender. These differences may be the result of differences in the social context and cultural meaning of health behaviors. Further research is needed to address gender disparities in health behaviors.

